# Multicomponent gas separation and purification using advanced 2D carbonaceous nanomaterials†

**DOI:** 10.1039/d0ra04286b

**Published:** 2020-06-25

**Authors:** Sayyed Jalil Mahdizadeh, Elaheh K. Goharshadi

**Affiliations:** Department of Chemistry and Molecular Biology, University of Gothenburg 405 30 Göteborg Sweden sayyed.jalil.mahdizadeh@gu.se; Department of Chemistry, Ferdowsi University of Mashhad Mashhad 9177948974 Iran

## Abstract

Multicomponent gas separation and purification is an important pre- or post-processing step in industry. Herein, we employed a multiscale computational approach to investigate the possibility of multicomponent low-weight gas (H_2_, O_2_, N_2_, CO_2_, CH_4_) separation and purification using novel porous 2D carbonaceous nanomaterials, namely Graphdiyne (GD), Graphenylene (GN), and Rhombic-Graphyne (RG). The dispersion-corrected plane-wave density functional theory (DFT) calculation combined with the Climbing Image Nudged Elastic Band (CI-NEB) method was employed to study the gas/membrane interaction energy and diffusion barrier of different gases passing through the geometrically optimized membranes. The results from CI-NEB calculations were then fitted to the Morse potential function to construct a bridge between quantum mechanics calculations and non-equilibrium molecular dynamics (NEMD) simulation. The selectivity of each membrane for all binary mixtures was calculated using the estimated diffusion energy barriers based on the Arrhenius equation. Finally, a series of extensive NEMD simulations were carried out to evaluate the real word and time dependent separation process. According to the results, CH_4_ molecules can be completely separated from the other gases using a GD membrane, O_2_ molecules from CH_4_, N_2_, and CO_2_ by a GN membrane, and H_2_ molecules from all other gases using a RG membrane.

## Abstract 2

Multicomponent gas separation and purification is an important pre- or post-processing step in industry.

## Graphical abstract

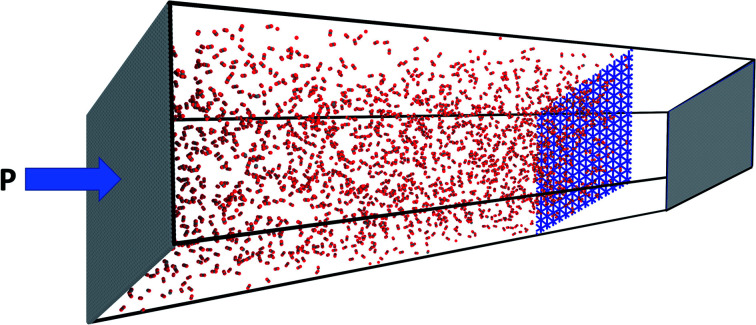

## Introduction

Multicomponent gas separation and purification is a vital pre- or post-processing step in many applications from large scale industrial ones to laboratorial low scale experiments.^1^ From the practical point of view, the efficient separation of low weight gases (H_2_, O_2_, N_2_, CO_2_, CH_4_) is of great significance because many of the light gases are feeds for many industrial processes.^1^ For instance, H_2_ can be separated from the other ingredients of syngas (CO and CO_2_) to employ it as a green energy carrier with zero environmental footprint.^2^ O_2_ separation from the air is strongly encouraged by exponentially increasing demands in medical and industrial sectors.^3^ CO_2_ is one of the most significant greenhouse gases that is emitted to the Earth's atmosphere as a consequence of the world's dependence on fossil fuels which leads to severe climate changes and global warming.^2^ Therefore, CO_2_ capture, separation, and recycling from the combustion products of fossil fuels have great advantages for environment and related industries.^4^ In natural gas sweetening process, CH_4_ molecules separate from other contaminants such as CO_2_ and H_2_S.^5^ N_2_ is an important gas which is widely used as inert atmosphere in air-sensitive chemical reactions and cooling media because of its suitably low boiling point (77.3 K).^6^

Among various gas separation methods, membrane technique provides several advantages such as high energy efficiency, facile operation, easy maintenance, and low investment cost.^7^ The membrane separation is typically referred to separation technology based on a semipermeable or selective membrane.^2,8^ The membrane performance in a gas separation process is basically determined with two parameters, selectivity and permeability. Selectivity is the capability of a membrane to selectively separate a desired molecule from a mixture. Whereas, permeability shows the membrane's productivity per unit time.^9^ An ideal membrane, for gas separation purposes, should have a low diffusion barrier for a specific type of molecule (permeability) and high diffusion barrier for other components within the gas mixture (selectivity). Apparently, a membrane with high selectivity usually suffers from low permeability, and *vice versa*.^10^ Therefore, there is always an intercommunication between selectivity and permeability of a membrane.

Traditional membranes for gas separation, like polymers,^11^ metals,^12^ zeolites,^13^ silica,^14^ and metal organic frameworks^15^ do not possess both high permeability and selectivity. Carbonaceous materials can be considered as very promising membranes in gas separation processes, since carbon is an abundant element and its allotrope's production techniques have been widely evaluated.^9^ It has been proven that the membrane permeability inversely correlates with its thickness.^16^ Therefore, porous 2D graphene-based nanomaterials, with one-atom thickness, have fascinated a great attention as efficient membranes for gas and liquid separation and purification processes.^17–21^

The pristine graphene is totally impermeable to any kind of gases even tiny He molecules.^22^ Hence, making in-plane pores in graphene sheets is necessary to attain molecular permeability. However, carving perfect and precise nanopores at large density level on a graphene sheet is extremely tricky and needs advanced breakthroughs in nano-scale manufacturing technologies.^23^ Therefore, finding novel 2D membranes with intrinsic uniform nanopores with specific architecture is essential as an alternative route. Graphenylene is an interesting allotrope of graphene with all the sp^2^-hybridized carbon atoms which was firstly suggested by Balaban *et al.*^24^ Graphenylene has attracted a great attention because of its thermal and mechanical stability and especially periodic unique pore architecture.^25–27^ Recently, some research groups could successfully synthesize graphenylene.^3,28^ Similar to graphene and graphenylene with purely sp^2^-hybridized network, other advanced 2D carbonaceous nanomaterials with successive sp^2^- sp-hybridized carbon atoms have been hypothesized theoretically.^29^ For example, the graphyne family can be built by replacing 1/3 of C–C bonds in graphene with n-acetylene linkages (–C≡C–) (*n* = 1, 2, 3, …) which would produce graphyne, graphyne-2 (graphdiyne), graphyne-3, *etc.*, respectively.^30^ Interestingly, some experimental techniques have been employed to successfully produce graphynes family.^31–33^ On the other hand, replacement of 2/3 of C–C bonds in graphene with acetylene linkage will produce a new 2D layered carbon allotrope called rhombic-graphyne.^34^

Due to the precise and uniform pore structure of these 2D nanomaterials, they are considered as promising ideal membrane for gas separation and purification.^35^ Jiao *et al.*^36^ evaluated the potential application of graphdiyne as membrane to separate H_2_ from syngas. According to their findings, graphdiyne shows a H_2_ permeability about 10^4^ times greater than that of porous graphene. Zhao *et al.*^37^ investigated the selective separation of different light gases by H-, O-, and F-substituted graphdiyne using computational approaches. They found that O- and F-substituted graphdiyne could efficiently separate CH_4_ and N_2_ gases. Cranford *et al.*^38^ estimated the flux of H_2_ passing through the graphdiyne membrane to be 7–10 g cm^−2^ s^−1^ from a gas mixture containing CH_4_ and CO molecules. Employing the first principle calculations, Zhang^35^ studied the H_2_ separation features of graphynes family over light gas molecules (*e.g.* CH_4_, N_2_, CO). According to their results, graphyne was not a suitable membrane for H_2_ separation because of its small pore size. However, graphdiyne demonstrated a high selectivity for H_2_ molecules (10^9^) over bigger molecules like CH_4_ but relatively low selectivity (10^3^) over smaller molecules. In addition, they showed that rhombo-graphyne has an extremely high selectivity for H_2_ molecules (10^16^) over other light gases. Zhang *et al.*^39^ showed that some graphyne derivatives, with pore sizes of 7 × 8 Å, could effectively blocks both di-branched and mono-branched pentane isomers. Using the dispersion-corrected DFT calculations, Zhu *et al.*^1^ estimated the separation performance of light gas mixtures *via* strained-control graphenylene. Their results indicated that applying lateral strain has a notable impact on the separation performance and selectivity of graphenylene membrane.

Herein, using dispersion corrected DFT calculations (DFT-D3) and non-equilibrium molecular dynamics simulation (NEMD), we have evaluated the selective separation performance of Graphdiyne (GD), Graphenylene (GN), and Rhombo-Graphyne (RG) for multicomponent mixture of light gases including H_2_, N_2_, O_2_, CO_2_, and CH_4_ molecules.

## Methods

### Quantum mechanics calculations

The quantum mechanics calculations were performed using Quantum ESPRESSO package.^40^ All geometry optimizations were carried out based on the periodic variable-cell plane-wave DFT calculations with the generalized gradient approximation (GGA) using Perdew–Burke–Ernzerhof (PBE) functional.^41^ The ultrasoft pseudopotential prepared through the Rappe–Rabe–Kaxiras–Joannopoulos scheme^42^ was used to model the ionic cores. The kinetic cutoff for charge densities and wave functions were defined to 4000 and 400 eV, respectively. The Brillouin zone integration was performed by an 8 × 8 × 1 Monkhorst–Pack grid.^43^ The convergence procedure was enhanced by applying Marzari–Vanderbilt cold smearing with 0.95 eV of broadening parameter.^44^ A vacuum gap with 20 Å thickness was considered in *z*-direction to minimize the interaction between periodic images. During the structural optimization process, the positions of all atoms in the unit-cell were fully relaxed until the convergence criteria of 1 × 10^−4^ eV for total energies and 1 × 10^−3^ eV Å^−1^ for forces were met. Also, the criterion for self-consistent field calculation was set to be 1 × 10^−5^ eV.

The Climbing Image Nudged Elastic Band (CI-NEB) method^45^ was used with dispersion-corrected DFT calculations (DFT-D3),^46^ as implemented in the Quantum ESPRESSO package, to investigate the minimum energy pathways (MEPs) of various gas molecules passing through the different membranes and to extract the interaction potential function parameters. The path threshold for CI-NEB calculations was set to 0.05 eV Å^−1^ and 20 points were defined to discretize the path.

### NEMD simulations

For non-equilibrium molecular dynamics (NEMD) simulation, the structurally optimized membranes with area of about 158 nm^2^ were placed in the middle of the permeate and feed sides. The feed side of the simulation box was filled up with 2000 gas molecules from each type, while the permeate side was set to be empty. Two rigid sheets of graphene were used as pistons to apply external pressure on the permeate and feed chambers. During the NEMD simulation, all the membranes were considered as rigid body because it has been stablished that the flexibility of the membrane has insignificant effect on the outputs.^47^ Also, it has been demonstrated that graphyne derivatives are much more rigid than other porous 2D materials like porous graphene and porous boron nitride membranes.^39^ Besides, the main goal of this study is to estimate the gas permeability at low pressure using linear interpolation of discrete data, where the possible membrane's distortion is at its minimum level.

All NEMD simulations were carried out by LAMMPS package.^48^ The velocity Verlet scheme was employed with time step of 0.5 fs to integrate the equation of atomic motions. The periodic boundary conditions were also applied in *X* and *Y* directions. The simulation box was first fully equilibrated for 2 ns in the NVT ensemble (Nose–Hoover thermostat) with a fixed 1 atm pressure exerted on both pistons along the *Z* direction. Afterwards, the production run was lunched in the NEMD scheme along with applying 100–700 MPa pressure on the feed's piston (Fig. 3). In NEMD scheme, exerting much higher pressure than that practically applied is utterly prevalent to elevate the signal-to-noise ratio and minimize thermal noises within a short timescale.^49^ To apply pressure (*P*), defined amount of force (*F*) was exerted on every individual atoms of the piston based on the equation, *F* = (*P* × *A*)/*n*, where *A* and *n* are the piston area and the number of atoms, respectively. Both pistons were free to move along the *Z* direction to reach the desired pressure.

The interaction energy between gas molecules and different membranes were extracted from quantum mechanics calculations and modeled using the Morse potential function as will discuss in the next section. The COMPASS force field^50,51^ was employed to describe both bonded and non-bonded interactions of gas molecules with 15 Å cutoff for van der Waals forces. Coulomb's law was employed for short-range coulombic interactions within a cutoff radius of 15 Å, while, PPPM technique^52^ was considered for long-range coulombic interaction.

## Results and discussion

### Quantum mechanics calculations

**Fig. 1:**
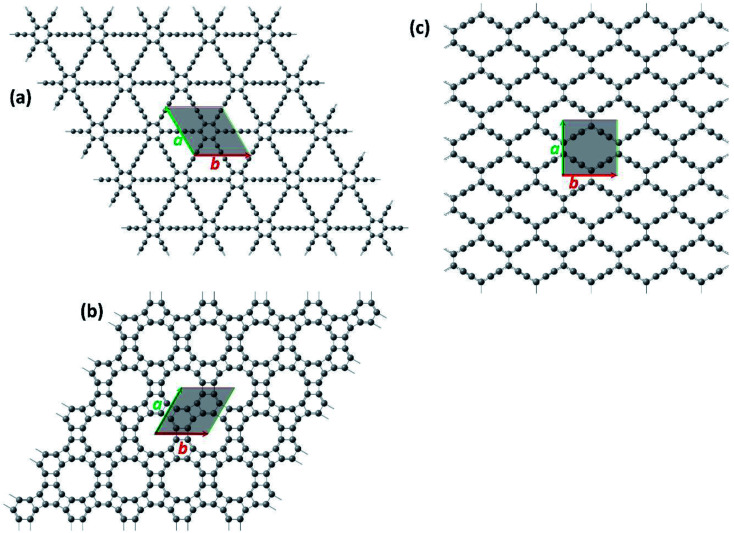
The optimized structure of (a) Graphdiyne (GD), (b) Graphenylene (GN), and (c) Rhombo-Graphyne (RG). The dashed area confined between two lattice vectors 
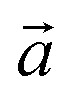
 and 
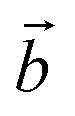
 represents the unit cells.

For the first step, the 2D structures of GD, GN, and RG were optimized by means of variable-cell DFT calculations. The optimized structures of the membranes used in the current study are shown in Fig. 1 where the dashed area confined between two lattice vectors 
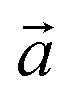
 and 
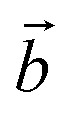
 represents the unit cells. The coordinates for optimized unit cells of different membranes are provided in the ESI.† The calculated cell lattice parameters are also presented in Table 1. As this table indicates, there is a very good agreement between lattice parameters calculated in this work and those reported in the literature. These results confirm that the calculated structures are accurate enough to provide a precise insight about the hole size and morphology of the membranes.

**Table 1:** The calculated cell lattice parameters of GD, GN, and RG from this work and those from the literature given in the parentheses

Membrane	*a* (Å)	*b* (Å)	*α* (°)
GD	9.43 (9.48),^53^ (9.39)^35^	9.43, (9.48),^53^ (9.39)^35^	120 (120)^53^
GN	6.74, (6.76)^53^	6.74, (6.76)^53^	60 (60)^53^
RG	6.97, (6.91)^1^	6.88, (6.84)^1^	90 (90)^1^

**Fig. 2:**
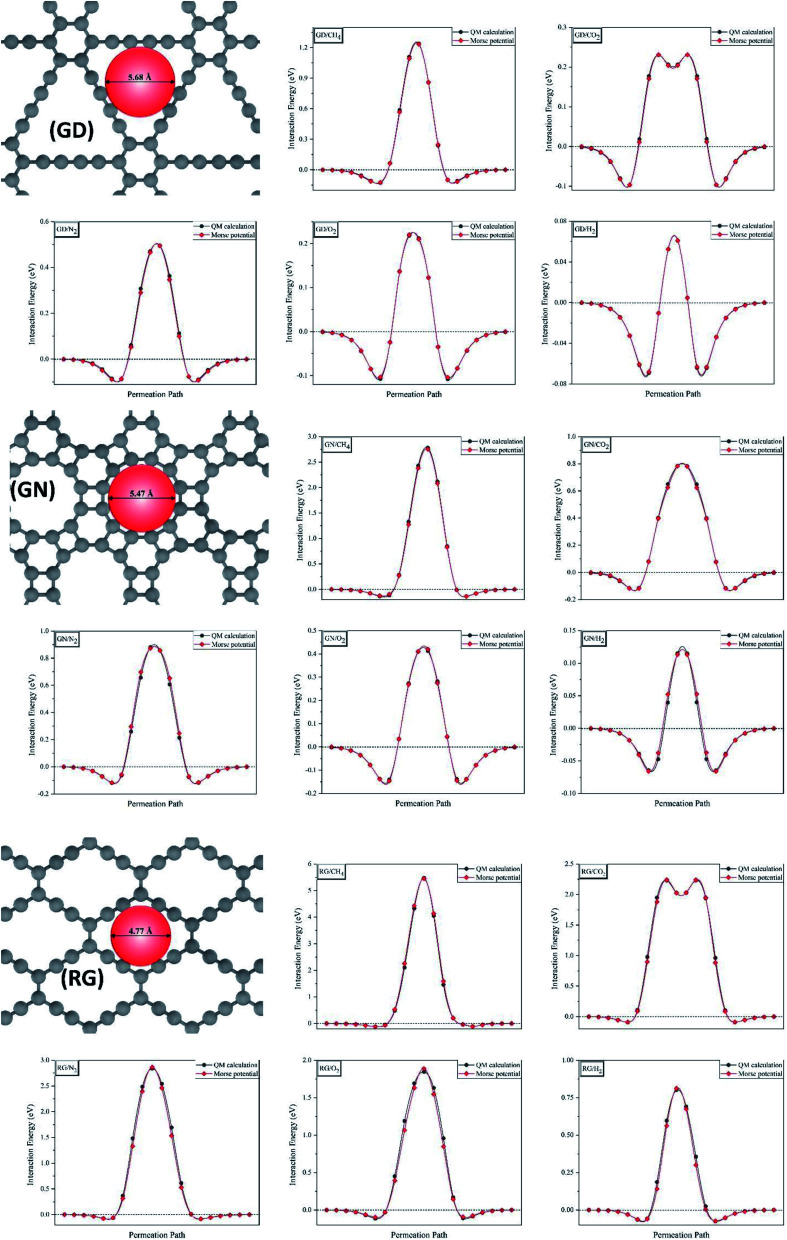
The interaction energy of various gas molecules passing through the different membranes calculated using CI-NEB and those fitted to the Morse potential. The van der Waals pore diameter of each membrane are presented by red spheres.

Afterwards, CI-NEB calculations were employed to investigate the MEPs of various gas molecules passing through the different membranes (Fig. 2) and to extract the interaction potential function parameters. The interaction energy between membrane surface and gas molecules, *E*, were calculated for 20 points which were used to discretize the MEP. Then, chi-square minimization technique was used to fit these points into the Morse potential (eqn (1)) by generalized reduced gradient algorithm.^54^

1 *E* = *D*_0_[e^−2*α*(*r*−*r*_0_)^−2e^−*α*(*r*−*r*_0_)^]

where *r*_0_, *D*_0_, and *α* are equilibrium distance (Å), well's width controller (Å^−1^), and potential well depth (eV), respectively. *r* is the distance between each atom of the adsorbate molecule and each carbon atom of the membrane. In practice, the distance between all atoms of the adsorbate molecule and all carbon atoms of the membrane were calculated at every 20 points of MEP. Then, the interaction energy calculated from the DFT CI-NEB was fitted into the summation over all the pairwise interactions. During the CI-NEB calculations and minimization process, the membrane was considered big enough to make it possible to sample all pairwise interactions within the cutoff radius of 10 Å, *i.e.* the total interaction energy doesn't change for a bigger membrane.

Fig. 2 shows the interaction energy of various gas molecules permeating through the different membranes calculated using CI-NEB and those fitted to the Morse potential. As this figure shows, the Morse potential fits very good at both attraction and repulsion regions. Table 2 compares the energy barriers for different gas molecules calculated using CI-NEB and those predicted from Morse potential. As one can see from Table 2, the difference between two energy barriers is less than 2% for all diffusing gas molecules. According to these results, the Morse potential could precisely reproduce the interaction energies and could be considered as a perfect bridge between quantum mechanics calculations and molecular dynamics simulation in this work. It has been approved that Morse potential can accurately describe the non-bonded interactions calculated by high level quantum mechanics techniques.^55^ The Morse potential parameters for interaction between various gas molecules and different membranes are shown in Table S1.†

**Table 2:** Energy barriers (eV) for gas molecules passing through the different membranes calculated using QM and those predicted from Morse potential and the absolute relative difference between these two values (Rel.)

	GD			GN			RG		
	Morse	QM	Rel. (%)	Morse	QM	Rel. (%)	Morse	QM	Rel. (%)
CH_4_	1.240	1.235	0.4	2.781	2.754	1.0	5.475	5.451	0.4
CO_2_	0.231	0.230	0.4	0.784	0.782	0.3	2.232	2.240	0.3
N_2_	0.494	0.494	0.0	0.880	0.872	0.9	2.838	2.864	0.9
O_2_	0.218	0.221	1.4	0.412	0.415	0.7	1.855	1.888	1.7
H_2_	0.061	0.061	0.0	0.115	0.113	1.8	0.801	0.812	1.3

The membrane selectivity for one gas (Gas_1_) over other gases (Gas_2_) can be estimated based on the Arrhenius equation:^52^2
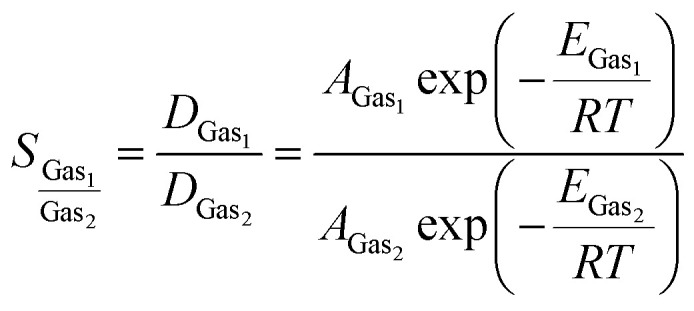
where *A*, *D*, *T* and *E* are the diffusion pre-factor, diffusion rate, absolute temperature, and diffusion energy barrier, respectively. Assuming that the passing-through processes of all gases follow the Arrhenius equation with the same pre-exponential factors^39^ and *T* = 300 K, the selectivity of membranes for each gas pairs can be calculated. The results are presented in Table 3. According to the selectivity results, the possibility of separating a mixture of gases can be examined by means of new membranes introduced here: For GD, the highest selectivity values belong to gas/CH_4_ pairs (gas = H_2_, O_2_, CO_2_, and N_2_) which implies CH_4_ molecules can be separated from the mixture using GD as membrane. For GN, the separation selectivity values for gas/CO_2_ and gas/N_2_ (gas = H_2_ and O_2_) are also high. Therefore, GN membrane seems to be able to separate H_2_ and O_2_ from the remaining gas mixture. On the other hand, the selectivity of H_2_/O_2_ on RG is high enough (1 × 10^18^) to anticipate that RG membrane can separate H_2_ and O_2_ molecules. However, using the Arrhenius equation to calculate the membrane selectivity is just an estimation because there are two major approximations: (1) the driving force for diffusion barrier is electronic energy not enthalpy, (2) the entropy contribution is included within the pre-factors which were assumed to be identical for each gas. Therefore, a series of extensive NEMD simulation were conducted to gain more realistic insight regarding the separation selectivity and gas permeation through the various membranes.

**Table 3:** The selectivity of different membranes for each gas pairs

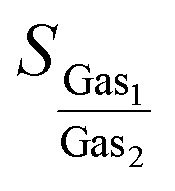	GD	GN	RG
H_2_/CH_4_	5 × 10^19^	2 × 10^44^	9 × 10^77^
H_2_/CO_2_	7 × 10^2^	2 × 10^11^	1 × 10^24^
H_2_/N_2_	2 × 10^7^	6 × 10^12^	3 × 10^34^
H_2_/O_2_	5 × 10^2^	1 × 10^5^	1 × 10^18^
O_2_/CH_4_	1 × 10^17^	2 × 10^39^	7 × 10^59^
O_2_/CO_2_	1.4	1 × 10^6^	8 × 10^5^
O_2_/N_2_	4 × 10^4^	5 × 10^7^	2 × 10^16^
CO_2_/CH_4_	8 × 10^16^	1 × 10^33^	9 × 10^53^
CO_2_/N_2_	3 × 10^4^	32.5	3 × 10^10^
N_2_/CH_4_	3 × 10^12^	4 × 10^31^	3 × 10^43^

### Non-equilibrium MD simulation

**Fig. 3:**
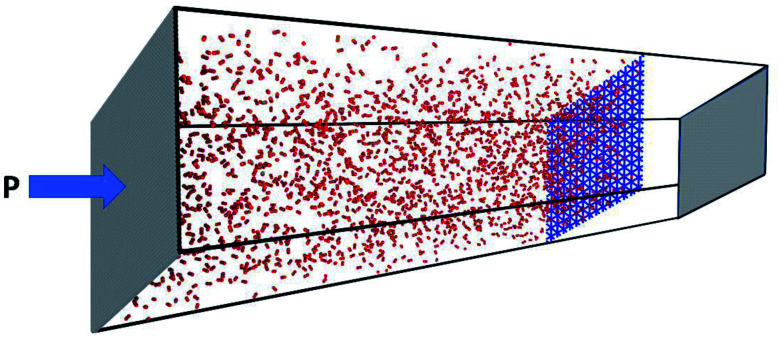
Snapshot of the simulation box after the initial 2 ns equilibrium stage. Two pistons and membrane are illustrated in grey and blue, respectively.

To gain realistic insight about selectivity and gas permeability of different membranes, a series of NEMD simulation were performed as describes in computational details section. Fig. 3 shows a snapshot of the simulation box after 2 ns equilibration, where two pistons (graphene sheets) and membrane (GD) is illustrated in grey and blue respectively. The NEMD simulation results approved that none of the membranes are permeable for CH_4_ molecules. On the other hand, GN and RG are impermeable for N_2_ and CO_2_ molecules, while they can pass through the GD membrane.

**Fig. 4:**
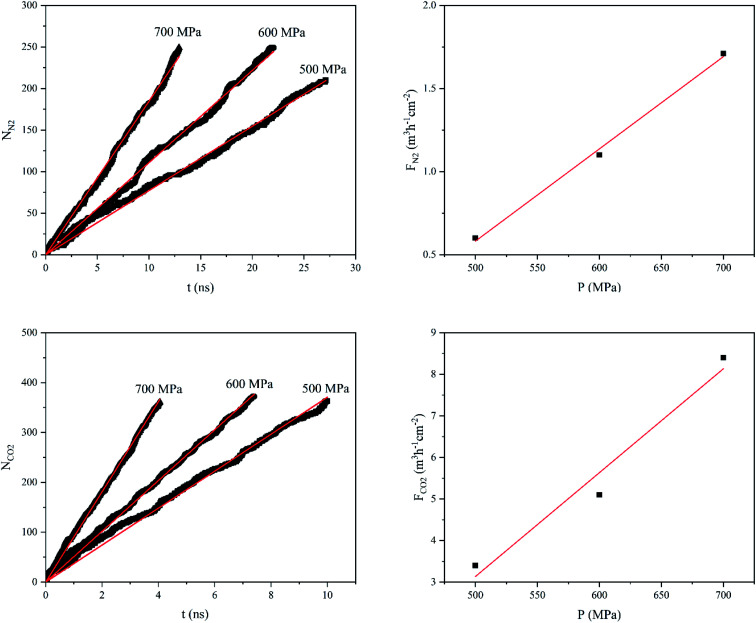
Number of N_2_ and CO_2_ molecules (*N*_gas_) passing through the GD a function of time (*t*), and gas flux (*F*_gas_) as a function of exerted pressure (*P*).

Fig. 4 shows the number of gas molecules (*N*_gas_, gas = N_2_ and CO_2_) passing through the GD as a function of applied pressure (*P*) and time (*t*). While both *N*_N_2__ and *N*_CO_2__ increase almost linearly with time, the rates of increase, at constant pressure, is much higher for CO_2_ gas. For example, at 500 MPa, *N*_N_2__ and *N*_CO_2__ reach to 200 after 26 and 5.5 ns, respectively. In addition, Fig. 4 illustrates the gas flux (*F*_gas_, gas = N_2_ and CO_2_) as a function of applied pressure. As one can see, there is also a linear correlation between gas flux and applied pressure. Hence, the gas permeability can be estimated from the slope of the linear plot *F*_gas_*vs. P*. The gas permeability values of GD membrane for CO_2_ and N_2_ gases were calculated to be 25.1 and 5.5 L h^−1^ cm^−2^ MPa^−1^ (at STP), respectively.

**Fig. 5:**
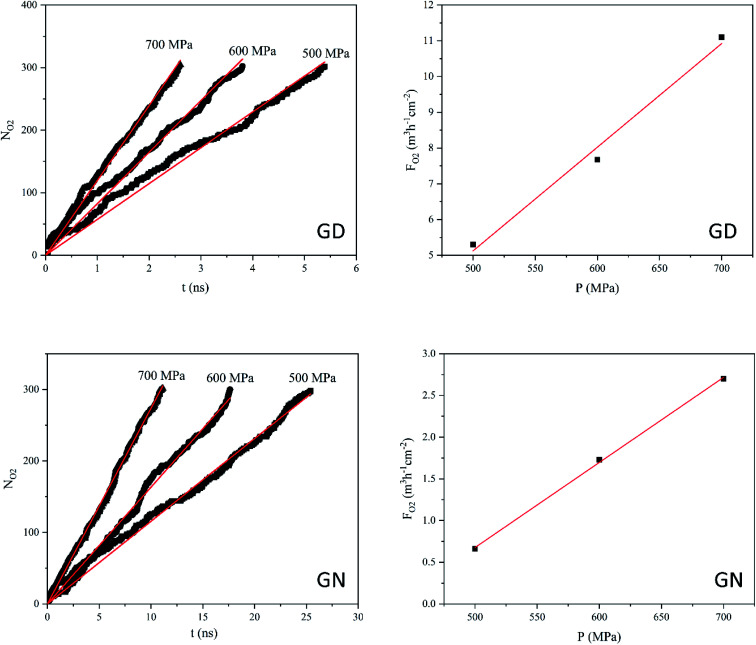
Number of O_2_ molecules (*N*_O_2__) passing through the GD and GN membranes as a function of time (*t*) and, and O_2_ flux (*F*_O_2__) as a function of exerted pressure (*P*).

According to the NEMD simulation results, oxygen molecules are able to diffuse through the GD and GN membranes but not RG. Fig. 5 shows the *N*_O_2__ and *F*_O_2__ values of GD and GN membranes as a function of applied pressure and time. The number of oxygen molecules passing through both GD and GN membranes increases almost linearly with time. However, at the constant pressure, *N*_O_2__ for GD is much higher than that of GN. For instance, at 500 MPa, *N*_O_2__ reaches to 300 after 5.4 and 25 ns for GD and GN membranes, respectively. The O_2_ gas permeability values were estimated to 29.3 and 9.8 L h^−1^ cm^−2^ MPa^−1^ (at STP) for GD and GN membranes, respectively.

**Fig. 6:**
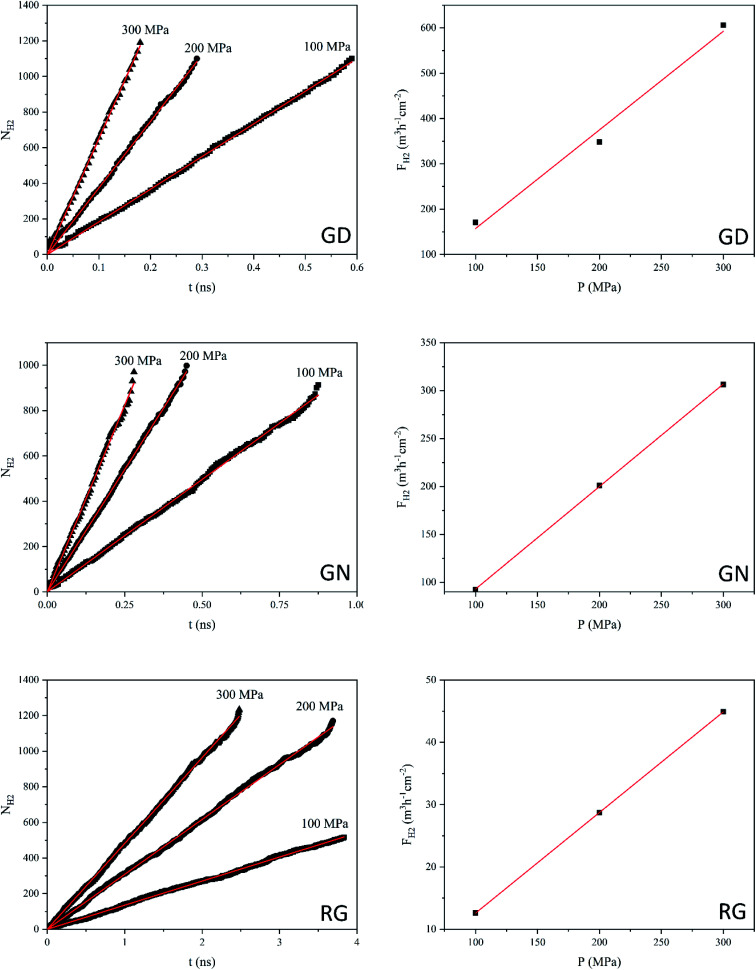
Number of H_2_ molecules (*N*_H_2__) passing through the GD, GN, and RG membranes as a function of time, and H_2_ flux (*F*_H_2__) as a function of exerted pressure (*P*).

Hydrogen molecules, due to the smallest size, can pass through all different types of membranes. Fig. 6 shows the *N*_H_2__ and *F*_H_2__ values of GD, GN, and RG membranes. NEMD simulations show that, at the constant applied pressure, the number of H_2_ molecules diffusing through the RG is significantly lower than those of GD and GN membranes. For example, at 300 MPa, the time elapsed for *N*_H_2__ to reach 1000 was 0.15, 0.26, and 2.0 ns for GD, GN, and RG membranes respectively. The calculated H_2_ gas permeability values were calculated to be 2180.1, 1070.5, and 160.2 L h^−1^ cm^−2^ MPa^−1^ (at STP) for GD, GN, and RG membranes, respectively.

The NEMD simulation results gave us the following insights: (1) methane molecules can be separated from the other gases (*i.e.* H_2_, O_2_, CO_2_, N_2_) using the GD as membrane (2) none of the membranes can completely separate CO_2_ and N_2_ molecules. However, the permeability of CO_2_ molecules through the GD membrane is ∼5 times greater than that of N_2_ molecules. (3) O_2_ and H_2_ can be separated from other gases by means of GN membrane. (4) H_2_ and O_2_ molecules can be separated perfectly using RG membrane. (5) the NEMD simulation results are generally consistent with the selectivity data calculated from Arrhenius equation. However, after analyzing the NEMD data and comparing the results with Arrhenius selectivity values, the limitation of the Arrhenius equation for prediction of the true selectivity was clearly revealed. For example, RG membrane is totally impermeable for all gases except H_2_, but Arrhenius predicts a very high selectivity for O_2_/CH_4_ (∼10^60^), CO_2_/CH_4_ (∼10^54^), and N_2_/CH_4_ (∼10^43^). It is because the selectivity value based on the Arrhenius equation depends on the difference between the diffusion barrier energies of each gas and not the absolute values. Therefore, a membrane can be impermeable for both gases (with very different barrier energies) while the Arrhenius shows a very high selectivity.

## Conclusions

Herein, we employed multiscale computational approach, combining plane-wave DFT calculations and extensive NEMD simulation, to investigate the possibility of multicomponent low weight gas (H_2_, O_2_, N_2_, CO_2_, CH_4_) separation and purification using novel porous 2D carbonaceous nanomaterials, namely Graphdiyne (GD), Graphenylene (GN), and Rhombic-Graphyne (RG). The results indicated that CH_4_ molecules are not able to pass through any of these membranes while CO_2_ and N_2_ molecules can just pass through DG membrane. The calculated permeability values of GD membrane for CO_2_ and N_2_ molecules are 25.1 and 5.5 L h^−1^ cm^−2^ MPa^−1^ (at STP), respectively. O_2_ molecules can pass through GD and GN membranes with the corresponding permeability values of 29.3 and 9.8 L h^−1^ cm^−2^ MPa^−1^ (at STP), respectively. On the other hand, H_2_ molecule can diffuse through all membranes with estimated permeability values of 2180.1, 1070.5, and 160.2 L h^−1^ cm^−2^ MPa^−1^ (at STP) for GD, GN, and RG membranes, respectively. This study shows that CH_4_ molecules can be completely separated from the other gases using GD membrane, O_2_ molecules from CH_4_, N_2_, and CO_2_ by GN membrane, and H_2_ molecules from all other gases using RG membrane. However, it seems complete separation of CO_2_ and N_2_ molecules is not possible with three membranes studied here. According to the results, graphdiyne, graphenylene, and rhombic-graphyne nanomaterials are promising membranes for multicomponent gas separation and purification.

## Conflicts of interest

There are no conflicts to declare.

## Supplementary Material

RA-010-D0RA04286B-s001

RA-010-D0RA04286B-s002

RA-010-D0RA04286B-s003

RA-010-D0RA04286B-s004
