# Supplementary material for: Multicomponent gas separation and purification using advanced 2D carbonaceous nanomaterials
Source: RSC Adv. 2020 Jun 25;10(41):24255–64. doi: 10.1039/d0ra04286b (PMC9055103; doi:10.1039/d0ra04286b)
Supplement: RA-010-D0RA04286B-s004 [file RA-010-D0RA04286B-s004.pdf]

Electronic Supplementary Material (ESI) for RSC Advances.
This journal is © The Royal Society of Chemistry 2020

Online Supporting Information for:

Multicomponent Gas Separation and Purification Using Advanced 2D Carbonaceous Nanomaterials

Sayyed Jalil Mahdizadeh[1,2,*], Elaheh K. Goharshadi[2]

[1] Department of Chemistry and Molecular Biology, University of Gothenburg, 405 30 Göteborg, Sweden.

[2] Department of Chemistry, Ferdowsi University of Mashhad, Mashhad 9177948974, Iran.

*corresponding author: Sayyed Jalil Mahdizadeh

Email: sayyed.jalil.mahdizadeh@gu.se

Table S1. The Morse potential parameters for interaction between various gas molecules and different membranes.

Gas	*Interaction* [a]	D_0 (eV)	α (Å^{-1})	r_0 (Å)
CH_4	C_{CH4}_C_{GD}	0.0065	1.4652	3.8863
	H_{CH4}_C_{GD}	0.0060	1.4013	3.8359
	C_{CH4}_C_{GN}	0.0064	1.4051	3.8896
	H_{CH4}_C_{GN}	0.0012	1.6033	3.6265
	C_{CH4}_C_{RG}	0.0011	1.5342	3.6500
	H_{CH4}_C_{RG}	0.0013	1.5772	3.6400
CO_2	C_{CO2}_C_{GD}	0.0040	1.3800	3.7250
	O_{CO2}_C_{GD}	0.0040	1.5700	3.7326
	C_{CO2}_C_{GN}	0.0041	1.3960	3.7600
	O_{CO2}_C_{GN}	0.0044	1.5950	3.7541
	C_{CO2}_C_{RG}	0.0045	1.4202	3.7382
	O_{CO2}_C_{RG}	0.0044	1.7043	3.7300
N_2	N_{N2}_C_{GD}	0.0047	1.6050	3.7695
	N_{N2}_C_{GN}	0.0046	1.5050	3.7430
	N_{N2}_C_{RG}	0.0043	1.5700	3.8200
O_2	O_{O2}_C_{GD}	0.0052	1.5323	3.6829
	O_{O2}_C_{GN}	0.0057	1.4171	3.6369
	O_{O2}_C_{RG}	0.0059	1.5476	3.6642
H_2	H_{H2}_C_{GD}	0.0031	1.5635	3.5471
	H_{H2}_C_{GN}	0.0022	1.4700	3.5111
	H_{H2}_C_{RG}	0.0035	1.6500	3.4800

[a] A_X_C_Y notation indicates the pairwise interaction between A atom(s) of the gas X and C atoms of the membrane Y.

Optimized unit cell of different membranes

1- GD membrane

C	1.779210955	2.778515981	0.000000000
C	3.211214910	2.778318236	0.000000000
C	3.927146847	4.018497572	0.000000000
C	3.211162889	5.258558873	0.000000000
C	1.779235557	5.258697197	0.000000000
C	1.063281838	4.018571332	0.000000000
C	-0.325871955	4.018697640	0.000000000
C	-1.553739611	4.018653612	0.000000000
C	1.084679998	6.461666109	0.000000000
C	0.470274924	7.524764816	0.000000000
C	3.905769487	6.461568236	0.000000000
C	4.519831677	7.524879654	0.000000000
C	5.316150348	4.018424785	0.000000000
C	6.544121261	4.018511802	0.000000000
C	3.905460254	1.575333527	0.000000000
C	4.519090018	0.511712010	0.000000000
C	1.084577268	1.575524979	0.000000000
C	0.470824048	0.511916325	0.000000000

2- GN membrane

C	2.611942491	0.680392950	0.000000000
C	4.086933972	0.680330378	0.000000000
C	4.769519750	1.862409672	0.000000000
C	4.031874608	3.139866051	0.000000000
C	2.667133010	3.139849546	0.000000000
C	1.929635713	1.862332381	0.000000000
C	6.038710262	2.595096550	0.000000000
C	7.403674794	2.595200855	0.000000000
C	8.141059425	3.872605493	0.000000000
C	7.458585029	5.054681557	0.000000000
C	5.983552339	5.054658192	0.000000000
C	5.301082123	3.872629515	0.000000000

3- RG membrane

C	0.180790330	2.711154795	0.000000000
C	0.180223352	4.160662266	0.000000000
C	1.378184558	2.009183867	0.000000000
C	1.377070468	4.864160540	0.000000000
C	2.426010165	1.376427352	0.000000000
C	2.424455055	5.497917784	0.000000000
C	3.622876006	0.673377701	0.000000000
C	3.622185343	6.199860981	0.000000000
C	4.820218252	1.375435136	0.000000000
C	5.868311778	2.007762184	0.000000000
C	4.819463192	5.497112613	0.000000000
C	5.867228860	4.863980587	0.000000000
